# Reliability and validity of the Korean version of organizational justice questionnaire

**DOI:** 10.1186/s40557-018-0238-8

**Published:** 2018-04-23

**Authors:** Hanul Park, Kang-Sook Lee, Yong-Jun Park, Dong-Joon Lee, Hyun-Kyung Lee

**Affiliations:** 1 Department of Preventive Medicine, College of Medicine, The Catholic University of Korea, 222, Banpo-daero, Seocho-gu, Seoul, 06591 Republic of Korea; 2 Graduate School of Public Health, The Catholic University of Korea, Seoul, Republic of Korea

**Keywords:** Organizational justice, Reliability, Validity, Scale development

## Abstract

**Background:**

Many studies show that organizational justice (OJ) is related to psychological determinants of employee health. To prevent health problems related to OJ in Korean workplaces and to accurately measure OJ, we developed the Korean version of the Organizational Justice Questionnaire (K-OJQ) and assessed its validity and reliability.

**Methods:**

A questionnaire draft of the K-OJQ was developed using back-translation methods, which was preliminary tested by 32 employees in Korea. Feedback was received and the K-OJQ was finalized. This study used data from 303 workers (172 males, 131 females) in Korea using the K-OJQ, job stress, and lifestyle questionnaires.

**Results:**

Cronbach’s α coefficients of the internal consistency reliability was 0.92 for procedural justice and 0.94 for interactional justice. Factor analyses using SPSS 24 and Amos 23 extracted two expected factors, named procedural justice (7 items; range, 1.0–5.0) and interactional justice (6 items; range, 1.0–5.0) and showed a reliable fit (χ^2^ = 182; *p* = .000; GFI = .912; AGFI = .877; CFI = .965; RMSEA = .077). Furthermore, higher procedural justice and interactional justice levels were correlated with lower job demand (− 0.33; − 0.36), insufficient job control (− 0.36; − 0.41), interpersonal conflict (− 0.45; − 0.51), job insecurity (− 0.33; − 0.34), organizational system (− 0.64; − 0.64), and lack of reward (− 0.55; − 0.63).

**Conclusions:**

The K-OJQ was objectively validated through statistical methods.

**Electronic supplementary material:**

The online version of this article (10.1186/s40557-018-0238-8) contains supplementary material, which is available to authorized users.

## Background

The interaction between employees and supervisors at work is very important. Their relationship may be highly sensitive to resources/rewards and well-being. The workers’ belief that supervisors are considering their viewpoints, sharing information about decision-making, and treating them fairly and truthfully is a measure of justice in the workplace [1]. Unfairness of the organization has been associated with job dissatisfaction, reprisal, and lower levels of commitment to work [2] (O). Johnson, extending the job demand-control model, confirmed that low support from colleagues and supervisors was an adverse factor. According to this model, high demands, low job control, and low social support are assumed to be related to higher risk of illness. Additionally, when employees perceive higher organizational justice, they show greater motivation and cooperation, have better attitudes about work, and reduced psychological distress, sickness absence, and negative emotions [3–5]. Furthermore, studies in Europe and Japan have reported that organizational justice is closely related to physical and mental health problems [6, 7].

Research on organizational fairness has steadily increased over the last 50 years. A study by John Stacey Adams that facilitated the use of “justice issues” for the investigation and understanding of organizational life was published in 1965. For more than 50 years, experts’ efforts have confirmed that justice plays an important role in the socio-technical context [8]. Traditionally, organizational justice has been defined as the extent that the organizational environment is perceived as fair in accordance with established rules [9]. To be specific, the concept of organizational justice is the perception that employees are being treated fairly in the workplace [2], and can be further classified into procedural justice, interactional justice, and distributive justice [10]. Procedural justice refers to whether the opinions of employees affected by the decision-making process are consistently applied, accurate, correctable, unbiased, and ethical. Interactional justice refers to supervisors and employees treating each other with respect and consideration and how well the rationale for decisions is explained when procedures are implemented. Distributive justice has been shown to be strengthened when outcomes are consistent with implicit norms for allocation, such as equity or equality [10, 11]. These concepts of organizational justice may be confused with other psychosocial factors at work, because control over decision making (job control) is one of the components of procedural justice and supervisor support is closely related to interactional justice. However, organizational justice is different from other psychosocial factors because it is thought to explore the basic elements of the workplace social structure and resources that underlie supervisor support and job control [12].

A recent meta-analytic study in the United States found relationships between organizational justice and cardiovascular health for 290 public workers. Specifically, the study examined the mediating role of perceived organizational support in relation to organizational justice, heart rate, systolic blood pressure, and diastolic blood pressure, and showed that higher levels of both procedural justice and perceived organizational support were needed for reduced heart rate and reduced systolic and diastolic blood pressure [13]. In Japan, a prospective cohort study with a one-year observation period of 1588 employees was conducted and examined the effects of organizational justice on insomnia. Interactional justice was an associated factor for the onset and persistence of insomnia [14]. In addition, studies have demonstrated the relevance of health and fairness. Lack of procedural justice was associated with an increased risk of psychiatric disorders, self-rated health status, sickness absence, and psychological distress [15–17].

The organizational structure in many Asian countries is more vertical, collective, and hierarchy-oriented [18], compared to the occupational structure in European countries [19]. The role of cross-cultural differences in justice outcomes is an important area of organizational justice studies. Furthermore, cross-cultural perspectives on organizational justice are helpful in assessing the generalizability of organizational justice theories [20]. A modified version of Moorman’s scale has been used in occupational health studies to assess organizational justice [6, 10, 16, 17, 21, 22] (Additional file 1). However, this scale, which can assess organizational justice, has not been translated and developed for use with Korean workers, and its reliability and validity in this population has not been tested. When introducing and using instruments developed in foreign countries, it is necessary to conduct a conceptually and statistically rigorous verification procedure. It is very difficult to reproduce the structural characteristics of the original even if it is a well-translated instrument. Therefore, it is necessary to apply various procedures to verify that the translation is equivalent to the original [23]. We developed the Korean Version of Organizational Justice Questionnaire (K-OJQ) based on the modified English and Japanese versions of the Organizational Justice Questionnaire (J-OJQ). The aim of this study was to assess the internal consistency reliability, factor-based validity, and construct validity of this developed scale.

## Methods

### Instrument

#### Korean version of the organizational justice questionnaire (K-OJQ)

In our study, we translated a modified version of Moorman’s Organizational Justice Questionnaire (OJQ) and the Japanese version of the OJQ into Korean and examined its reliability and validity (Additional file 2). The K-OJQ was based on modified English [21] and Japanese versions of the OJQ [10] and consisted of seven items for procedural justice (PJ) and six items for interactional justice (IJ). The responses to each item were recorded on a five-point Likert scale (range: 1 = strongly disagree, 5 = strongly agree). The K-OJQ, which was translated from a modified version of Moorman’s OJQ [21] and the Japanese version of the OJQ [10], was developed by conducting a preliminary survey and translation–back translation. The translation focused on conceptual and vocabulary consistency, and was conducted in consideration of the cultural characteristics of Korea. Version 1 of the K-OJQ was completed by comparing a questionnaire that translated the modified English of the OJQ [21] into Korean and a questionnaire that translated the Japanese version of the OJQ [10] into Korean. We conducted a preliminary survey and received feedback from 21 medical staff (health screening specialists, dental hygienists, healthcare managers, and nurses) and 11 manufacturing staff and revised the translation. Specifically, the Korean translation of “viewpoint” in interactional justice item 1 was modified to “thinking methods” because it can be limited to “opinion” in Korean. In order to assess content validity, a professional group consisting of three professors of occupational environmental medicine was selected. The K-OJQ was verified by the expert group and all CVI exceeded 0.7, with the exception of item 1 in interactional justice.

#### Short form of the Korean occupational stress scale (KOSS-SF)

The KOSS-SF shortens the Korean Occupational Stress Scale (KOSS) [24], which was based on the Job Content Questionnaire [25], Effort-Reward Imbalance [26], Occupational Stress Index [27], and National Institute for Occupational Safety and Health model [28], and was developed in consideration of Korea-specific job stress factors. The KOSS-SF consists of seven subscales: job demand (4 items), insufficient job control (4 items), interpersonal conflict (3 items), job insecurity (2 items), organizational system (4 items), lack of reward (3 items), and occupational climate (4 items). Items were scored using four-point Likert scales (range: 1 = strongly disagree or strongly agree, 4 = strongly agree or strongly disagree). In the present study subjects, Cronbach’s α coefficients were 0.75, 0.7, 0.79, 0.62, 0.81, 0.84, and 0.78 for Job demand, Insufficient job control, Interpersonal conflict, Job insecurity, Organizational system, Lack of reward, and Occupational climate, respectively. Correlational analysis of the K-OJQ and KOSS-SF contributed to the criterion validity of the K-OJQ.

### Participants

This study used cross-sectional data obtained from 343 subjects (203 large, 140 medium or small sized enterprises workers) in Seoul and Gyeonggi Province, South Korea. Self-administered questionnaires were completed by subjects in April, 2017, and questionnaires were delivered to subjects directly or by mail. We excluded 40 subjects whose responses were missing in the questionnaire, and 303 subjects (172 males, 131 females) were analyzed. The purpose of this study was explained to all subjects, and written informed consent was obtained from participants. The design of this study was approved by the Institutional Review Board at The Catholic University of Korea (IRB approval number: MC17QASI0009).

### Statistical analysis

Statistical analyses were performed using SPSS 21.0 and AMOS 23.0 software. The general characteristics of the subjects were measured using frequency, percentage, mean, and standard deviation. Cronbach’s α was assessed to measure the internal consistency reliability. To assess structural validity, Bartlett’s test of sphericity and Kaiser-Meyer-Olkin measure of sampling adequacy (KMO) were performed on 303 subjects and exploratory factor analysis (EFA) was performed for the 13 OJQ items with maximum likelihood with Promax rotation. In the EFA, we extracted factors with values of 1 or more. To assess model fit, confirmatory factor analysis (CFA) was performed with the following statistical estimates: Goodness-of-Fit Index (GFI), Adjusted Goodness-of-Fit Index (AGFI), Comparative Fit Index (CFI), and Root Mean Square Error of Approximation (RMSEA). The acceptability of model fit was judged by the following criteria: GFI, AGFI, and CFI > 0.90 and RMSEA < 0.05 [29]. The criterion validity was assessed by Pearson’s correlations analysis between the K-OJQ and KOSS-SF.

## Results

### Sociodemographic characteristics

The mean age of the subjects was 35.5 (SD = 7.73), and “More than 12 years” was the largest educational category with 258 (85.1%). The number of daytime workers (91.1%) was more than that of shift workers (8.9%), and 256 (84.5%) of the subjects were regular workers (Table 1).

**Table 1:** Sociodemographic characteristics

Characteristics:	*N* = 303 (%)
Gender	
Men	172 (57)
Women	131 (43)
Age	
Years (mean ± SD)	35.54 ± 7.73
Educational status	
12 years or less	45 (14.9)
More than 12 years	258 (85.1)
Type of work	
Daytime	276 (91.1)
Shift work	27 (8.9)
Type of employment	
Regular	256 (84.5)
Temporary	47 (15.5)

### Internal consistency reliability

The mean value of PJ was 3.40, and IJ was 3.47. The internal consistency of Cronbach’s α showed a good internal consistency with 0.92 for PJ and 0.94 for IJ (Table 2).

**Table 2:** Mean value, standard deviation (SD), internal consistency of the OJQ scales

	*N* = 303	
Scale (number of items)	Mean ± SD^a^	Cronbach α
The Korea version of the organizational justice questionnaire:		
Procedural justice (7)	3.40 ± 0.87	0.92
Interactional justice (6)	3.47 ± 0.89	0.94
Occupational stress scale for Korean Employees:		
Job demand (4)	2.57 ± 0.53	0.75
Insufficient job control (4)	2.39 ± 0.52	0.70
Interpersonal conflict (3)	1.98 ± 0.54	0.79
Job insecurity (2)	2.22 ± 0.61	0.62
Organizational system (4)	2.24 ± 0.55	0.81
Lack of reward (3)	2.29 ± 0.60	0.84
Occupational climate (4)	2.21 ± 0.58	0.78

^a^*SD* standard deviation

### Structural validity

As a result of the EFA, 13 K-OJQ items were extracted into two factors, and these factors explained 68.84% of the total variance. The seven PJ items showed loading values of 0.64–0.88 on Factor 1 and the six IJ items showed loading values of 0.67–0.94 on Factor 2 (Table 3). In the CFA with two factors, the model fit showed good fit indices with GFI = 0.91, AGFI = 0.87, CFI = 0.96, and RMSEA = 0.07 (90% CI: 0.06–0.09) (Table 4).

**Table 3:** Exploratory factor analysis of 13 items of the Organizational Justice Questionnaire

		*N* = 303
Items	Factor 1	Factor 2
Procedural justice		
All sides affected by the decision are represented in decision making. (#3)	*0.88*	−0.06
Feedback is collected regarding the decision and its implementation. (#6)	*0.75*	0.06
People are provided opportunities to appeal or challenge decisions they find unsuccessful. (#2)	*0.75*	0.09
The concerns of all those affected by the decision are heard before decision making. (#5)	*0.74*	0.02
Decisions are made with consistency (the rules are the same for every employee). (#4)	*0.74*	0.11
Decisions are made based on accurate information. (#1)	*0.65*	0.13
It is possible to requests for clarification or additional information about the decision. (#7)	*0.64*	0.18
Interactional justice		
Our supervisor treats us with kindness and consideration. (#4)	−0.06	*0.94*
Our supervisor shows concern for our rights as an employee. (#5)	−0.01	*0.92*
Our supervisor takes steps to deal with us in a truthful manner. (#6)	0.12	*0.77*
Our supervisor considers our viewpoint. (#1)	0.09	*0.75*
Our supervisor is able to suppress personal biases. (#2)	0.12	*0.71*
Our supervisor provides us with timely feedback about the decisions and their implications. (#3)	0.17	*0.67*
Variance explained (%)	62.64%	6.20%

The maximum likelihood method and Promax rotation

**Table 4:** Confirmatory factor analysis of 13 items of the Organizational Justice Questionnaire

			*N* = 303
Items	Procedural justice	Interactional justice	*P*-value
Procedural justice #1	0.78		< .001
Procedural justice #2	0.82		< .001
Procedural justice #3	0.81		< .001
Procedural justice #4	0.83		< .001
Procedural justice #5	0.75		< .001
Procedural justice #6	0.81		< .001
Procedural justice #7	0.80		< .001
Interactional justice #1		0.83	< .001
Interactional justice #2		0.81	< .001
Interactional justice #3		0.81	< .001
Interactional justice #4		0.88	< .001
Interactional justice #5		0.90	< .001
Interactional justice #6		0.88	< .001
Fit indices:			
Goodness of fit index (GFI)			0.91
Adjusted goodness of fit index (AGFI)			0.87
Comparative fit index (CFI)			0.96
Root-mean square error of approximation (RMSEA) (90% CI)			0.07

Standardized estimate value and fit indices

### Criterion validity

The higher the PJ and the IJ levels, the lower the job demand (− 0.33, − 0.36), insufficient job control (− 0.36, − 0.41), interpersonal conflict (− 0.45, − 0.51), job insecurity (− 0.33, − 0.34), organizational system (− 0.64, − 0.64), lack of reward (− 0.55, 0.63), and occupational climate (− 0.50, − 0.55) (Table 5).

**Table 5:** Pearson’s correlation coefficients of Organizational Justice Questionnaire with Korean Occupational Stress Scale

			*N* = 303
	Procedural justice	Interactional justice	*P*-value
Job demand	−0.33	−0.36	< .001
Insufficient job control	−0.36	−0.41	< .001
Interpersonal conflict	−0.45	−0.51	< .001
Job insecurity	−0.33	−0.34	< .001
Organizational system	−0.64	−0.64	< .001
Lack of reward	−0.55	−0.63	< .001
Occupational climate	−0.50	−0.55	< .001

## Discussion

We translated the modified version of the OJQ [21] and Japanese version of the OJQ [10] into Korean and conducted a preliminary survey. Reliability and validity of the developed Korean version of the OJQ were examined using samples of Korean workers. The 13 items were extracted as two factors, procedural justice and interactional justice, which comprised the hypothesized structure. Both factors showed good internal consistency and had a correlation with the Korean Occupational Stress Scale.

The modified version of the OJQ [21] has been assessed for reliability and validity in several countries, and Cronbach’s α coefficients of the internal consistency reliability of the two factors in our study were 0.92 and 0.94, respectively. These values were higher than the Cronbach’s α coefficients of the G-OJQ [30] that was developed to measure justice in the German study. In the Japanese study, Cronbach’s α coefficients of men were 0.86 and 0.94 and of women were 0.85 and 0.94, respectively [10]. Procedural justice and interactional justice showed very high internal consistencies as in the studies in Japan and Germany.

The 13 K-OJQ items were extracted with 6 items of procedural justice and 7 items of interactional justice by exploratory factor analysis, and this structure was consistent with the structure of the modified version of the OJQ [21] and G-OJQ [30]. In the study in japan, the items showed the greatest factor loadings on two factors in men, and three factors in women, respectively [10]. In our study, when men and women were separated, and exploratory factor analysis was conducted, one factor was extracted for women and was not suitable for the modified version of the OJQ model. Thus, we did not separate men and women.

In the confirmatory factor analysis of all K-OJQ items, GFI, AGFI, CFI, and RMSEA indices reached the predetermined acceptable levels for the model. The indices of K-OJQ model fit were lower than those of the G-OJQ [30] and higher than those of the J-OJQ [10]. In content validity based on opinions of experts, item 1 of interactional justice was not high CVI, but criterion validity and construct validity were objectively validated.

The mean score of procedural justice in our study was higher than that in the Japanese [6] and Finnish studies. Additionally, the Finnish study’s mean score [16, 21, 22] was lower than that of the Japanese study. This result might be interpreted as being due to differences in the jobs of the surveyed subjects rather than as a difference in procedural justice between countries. The subjects of our research were white collar workers, whereas in the Japanese study, subjects were employees of a manufacturing factory [10]. Moreover, the Finnish study mainly assessed organizational justice in hospital employees [16, 17, 21, 22]. This finding might be interpreted to reflect a lack of decision making processes in workers at the hospital or the manufacturing factory than in office workers.

The mean score of interactional justice was higher in Korea than in Japan [12], and that in Finland [16, 17, 21, 22] was higher than in Korea and Japan. This finding was consistent with previous cross-cultural studies reporting that Korea, Japan, and China had substantially different attitudinal and behavioral patterns [31–33], because of East Asian characteristics of high collectivism and power distance [34]. Furthermore, this finding was consistent with previous studies reporting that many Asian countries have distinct features of being more vertical, collective, and workplace structure and hierarchy-oriented [18] than workplaces in European countries [19].

We predicted organizational justice to be related to job stress factors. Correlations between KOSS-SF [24] factors and K-OJQ factors provide support for the criterion validity of the K-OJQ. Higher procedural and interactional justice were correlated with lower levels of job demands, insufficient job control, interpersonal conflict, job insecurity, organizational system, lack of reward, and occupational climate. The job demands item, which indicates the degree of burden on the job, was not correlated with organizational justice in men in Kivimäki’s study [17] and Japanese studies [10]. However, higher organizational justice was correlated with lower job demands in men and women in our study. The results of our study were consistent with previous findings that higher levels of procedural justice were correlated with higher job control [10, 35]. In our study, insufficient job control was correlated with interactional justice rather than procedural justice. This result could be interpreted as reflecting the organizational culture of Koreans. The interpersonal conflict item measured the lack of support of supervisors and co-workers in the workplace. In the Japanese study, higher levels of coworker support and supervisor support items were correlated with higher procedural justice and interactional justice [10]. This finding was consistent with the result that justice reacted sensitively to relationships with peers and supervisor [26]. The job insecurity item in our study was similar to the job future ambiguity in the Japanese study. However, in the Japanese study, this item was not correlated with interactional justice. Organizational system items measure the overall characteristics of an organization, such as its operating systems, resources, conflicts, promotion, and rational communication. In our study, this item showed a greater correlation with organizational justice than other items, and this result may mean that organizational justice was more affected by the group than individuals. This finding was consistent with the study of Lind reporting that “most of the potential information about the fairness of any given authority or institution lies in collective, not personal, experience” [36]. In the Japanese study, higher levels of procedural and interactional justice were correlated with lower effort-reward imbalance [10]. In Moorman’s study [1] higher levels of organizational justice were correlated with higher distribution justice this was consistent with our study’s finding and organizational justice and reward could be interpreted as a strong reaction to each other. The occupational climate item measures Korean collectivistic culture, irrational communication systems, informal work culture, and sexual discrimination.

Our study and the modified OJQ have several limitations. First, the modified OJQ does not distinguish between interactional justice and informational justice that is an aspect of interactional elements that assesses the level of explanation from the decision maker, and lacks a subscale to measure distributive justice [37]. Second, the OJQ might not measure all aspects of organizational justice in the Korean workplace. For instance, Koreans were more likely to compromise with their supervisors to solve an interpersonal conflict than Chinese and Japanese [38]. The Korean workplace culture has characteristics that distinguish it from that of other countries, and these aspects may not be included in the current concept of organizational justice. Third, scholars have elevated justice study from the individual level to the unit level [39]. Procedural justice may well explain organizational structure or resources, but interactional justice may not distinguish a resource from supervisor support given to an individual worker. Thus, there was a limitation to understanding organizational structure and task level work characteristics. Finally, our study sample was extracted from one particular company in Seoul and Gyeonggi Province, and the test-retest reliability and concurrent validity of the K-OJQ were not examined. Generalization of the findings should be done with caution.

## Conclusion

This study was meaningful in that we developed an instrument to measure organizational justice in the workplace considering the characteristics of work culture and the organization of Korea and tested the reliability and validity of the instrument. Our instrument may be used in future occupational health studies. Future studies are needed to test the reproducibility and applicability of our instrument in other working populations and settings.

## Additional files

Additional file 1: The modified organizational justice questionnaire. (DOC 33 kb)

Additional file 2: The Korean version of organizational justice questionnaire. (HWP 16 kb)

## Abbreviations

AGFI: Adjusted goodness-of-fit index
CFA: Confirmatory factor analysis
CFI: Comparative fit index
EFA: Exploratory factor analysis
GFI: Goodness-of-fit index
IJ: Interactional justice
J-OJQ: The Japanese versions of the Organizational Justice Questionnaire
KMO: Kaiser-Meyer-Olkin measure of sampling adequacy
K-OJQ: The Korean version of the Organizational Justice Questionnaire
KOSS-SF: Short Form of the Korean Occupational Stress Scale
OJ: Organizational justice
OJQ: The modified version of Moorman’s Organizational Justice Questionnaire
PJ: Procedural justice
RMSEA: Root Mean Square Error of Approximation;
