# Supplementary material for: Reliability and validity of the Korean version of organizational justice questionnaire
Source: Ann Occup Environ Med. 2018 Apr 23;30:26. doi: 10.1186/s40557-018-0238-8 (PMC5913812; doi:10.1186/s40557-018-0238-8)
Supplement: Supplementary file 1 — The modified organizational justice questionnaire. (DOC 33 kb) [file 40557_2018_238_MOESM1_ESM.doc]

**THE MODIFIED ORGANIZATIONAL JUSTICE SCALES WE HAVE USED (translated from Finnish):**

**Procedural justice** – The scale is based on the following statements about decision making at the workplace:

1. Decisions are made based on accurate information.

2. People are provided opportunities to appeal or challenge decisions they find unsuccessful.

3. All sides affected by the decision are represented in decision making.

4. Decisions are made with consistency (the rules are the same for every employee).

5. The concerns of all those affected by the decision are heard before decision making.

6. Feedback is collected regarding the decision and its implementation.

7. It is possible to requests for clarification or additional information about the decision.

**Response format:**

1 = strongly disagree,... 5 = strongly agree

**Interactional justice** - The scale is based on the following statements about the general behaviour of the respondent's supervisor:

1. Our supervisor considers our viewpoint.

2. Our supervisor is able to suppress personal biases.

3. Our supervisor provides us with timely feedback about the decisions and their implications.

4. Our supervisor treats us with kindness and consideration.

5. Our supervisor shows concern for our rights as an employee.

6. Our supervisor takes steps to deal with us in a truthful manner.

**Response format:**

1 = strongly disagree,... 5 = strongly agree
